# Assessment of the Sludge Reduction of the Metabolic Uncoupler 3,3′,4′,5-tetrachlorosalicylanilide (TCS) in Activated Sludge Culture

**DOI:** 10.3390/ijerph16101686

**Published:** 2019-05-14

**Authors:** Xiaofan Yang, Xiaoping Xu, Xueyu Wei, Jincheng Li, Jie Wan

**Affiliations:** College of Biological and Chemical Engineering, Anhui Polytechnic University, Wuhu 24100, China; xiaofan108@ahpu.edu.cn (X.Y.); wxyu1027@126.com (X.W.); wjxfy108@126.com (J.L.); valleyvan.1983@aliyun.com (J.W.)

**Keywords:** sludge reduction, metabolic uncoupler, TCS, ectronic transport system activity, EPS, DNA

## Abstract

Batch experiments were completed to assess the sludge reduction of the metabolic uncoupler 3,3′,4′,5-tetrachlorosalicylanilide (TCS). The effects of various TCS concentrations on sludge yield were evaluated and the mechanisms associated with sludge reduction were assessed. We discovered that TCS addition resulted in a reduction in sludge. Furthermore, a low dose of TCS (≤3 mg/L) resulted in a slight reduction in the efficiency of the wastewater treatment system, while >3 mg/L TCS reduced matrix removal efficiency, with an especially remarkable inhibition effect on ammonia removal. An increase in TCS addition was associated with a gradual decrease in both the electron transport system (ETS) activity and the specific cellular ATP (SATP) in the TCS system. It was demonstrated that TCS plays an important role in metabolic uncoupling. However, with the addition of TCS, both contents and compositions were increased, and the protein content increased more than polysaccharide production in extracellular polymeric substances (EPS). At TCS concentrations of ≤3 mg/L, DNA content was stable, but it increased rapidly from 4.97 mg/L to 15.34 mg/L as the TCS concentration was elevated from 6 mg/L to 12 mg/L. This implied that the mechanisms of sludge reduction were different for different TCS concentrations, including uncoupling metabolism, maintenance metabolism and lysis–cryptic growth.

## 1. Introduction

Conventional activated sludge is a broadly utilized process in both municipal and industrial wastewater treatment plants (WWTPs). One disadvantage of this process relates to the significant quantities of excess sludge that are generated, which in some cases are as high as 50–60% of the total operational expenses of the wastewater treatment plants [1]. Post-treatment is thus presently broadly used for the disposal of activated sludge, which reduces the costs of transportation and further treatment. As a result of the great operational complexity and cost of the post-treatment processes, there has been an increased focus on the use of in situ excess sludge reduction processes, as they can lower excess sludge production at the origin [2,3,4]. In this regard, different approaches of microbiological or chemical processes have been employed, such as lysis–cryptic growth [5], maintenance metabolism [6], predation on bacteria [7] and uncoupled metabolism [8,9,10]. Of these techniques, uncoupled metabolism facilitated by the addition of metabolic uncouplers has been shown to be an incredibly effective technique.

Of the above-mentioned in situ excess sludge reduction technologies, the metabolic uncoupler is most widely accepted, since it can seamlessly feed into the aeration tank of a wastewater treatment plant, which avoids the necessity to change the conventional wastewater treatment process; because the metabolic uncoupler is usually a lipophilic soluble acid, it can transport protons across the cell membrane and result in partial decomposition due to catabolism and anabolism [11,12,13,14,15], which brings about reduced production of bacterial cell mass [16]. A variety of metabolic uncouplers, including 2,4-dinitrophenol (dNP), para-nitrophenol (pNP), 2,4-dichlorophenol (dCP), and 3,3′,4′,5-tetrachlorosalicylanilide (TCS), have been used in the reduction of sludge yield [13,17,18] in sequencing batch reactors and batch experiments. TCS has been applied in the production of soaps, body washes, shampoos, and other products [19], and is believed to constitute a mild and environmentally friendly metabolic uncoupler [18]. TCS effectively reduces additional sludge production, including at very low concentrations. A TCS quantity of 0.8–1.0 mg/L in a batch system is reported to result in a 40% depletion in sludge yield [17].

The majority of studies on metabolic uncouplers have been aimed at determining their capacity to decrease biomass production and the impacts of chemical uncouplers on substrate removal efficiency [11,12,13,14,15]. In contrast with the widespread evaluation of the utilization of metabolic uncouplers, the enhancement mechanisms of sludge depletion by uncouplers have not been systematically analyzed to date. Additionally, the impact of chemical uncouplers on the inhibition of microorganism activity has not been comprehensively assessed. Moreover, what the remaining part of the substrate is used for and where the energy is utilized remain unknown. Several recent studies have evaluated the connection among microbial offerings and sludge depletion with the addition of metabolic uncouplers. Soluble microbial products (SMP), which consist of proteins, polysaccharides, humic acid, DNA, and lipids, are required to offer a safety shield for the bacteria within the activated sludge.

In the current evaluation, the effects of TCS addition on extra sludge yield and nutrient elimination were evaluated. Furthermore, the impact of TCS on microbial activity inhibition and energy distribution was also investigated. We believe that the findings will help improve our comprehension of the reaction of activated sludge systems to metabolic deodorants, such as TCS in biological wastewater treatment.

## 2. Materials and Methods

### 2.1. Activated Sludge and Wastewater

The activated sludge utilized in the experiments was sampled at a local municipal wastewater treatment plant, following which it was sieved (2-mm mesh) to eliminate coarse pieces. Prior to experimentation, the sludge was cultivated at room temperature in six identical sequencing batch reactors (SBR), each with a working volume of 3.2 L. The SBRs were operated for four cycles daily, and every cycle lasted 6 h. There were five stages in each cycle: fill (30 min), react (4 h with aeration), settle (1 h), draw (20 min), and idle (10 min). For the filling, drawing, and aeration reactions, the operating cycle was controlled automatically with a time device. The dissolved oxygen (DO) concentration was maintained greater than 4 mg/L, and the mixed liquid suspended solid (MLSS) level was retained at around 2500 mg/L by removing any extra sludge. The sludge was cultivated for 40 days in the absence of TCS, following which different concentrations (0, 0.6, 1, 1.5, 3, 6, and 12 mg/L) of TCS were added.

The synthetic wastewater utilized for cultivation comprised sodium acetate and sucrose as the only carbon source for bacterial growth; ammonium chloride was used as the nitrogen source as well as additional trace elements, as shown in Table 1. Additionally, the wastewater was supplemented with 0.1 mol/L HCl or NaOH to maintain a pH of approximately 7.0.

### 2.2. TTC-ETS Activity and SATP Assay

The homogenous sludge samples were obtained every three days to calculate the electron transport system (ETS) activity. 2,3,5-triphenyltetrazoliun chloride (TTC) is a kind of oxidoreduction dye used to detect the activity of sludge ETS. TTC-ETS was calculated to evaluate the impacts of TCS on ETS activities, and MLSS was utilized as an indicator of the biomass of sludge microorganisms. TTC-ETS activity was calculated based on Kenner Ahmed [20], as modified by Martinez [21]. TTC-ETS activity was calculated using Equation (1) [22], where TTC-ETS is the electron transport system activity (μg mg^−1^ h^−1^), D_485_ is the absorbance at 485 nm, V is the extract volume (mL), k is the slope of the standard curve (mL μg^−1^), W is the dry weight of the activated sludge (mg), and t is the incubation time (h).

(1) $$TTC\text{-}ETS=\frac{D_{485}V}{kWt}$$

Cellular ATP was removed using the altered procedure of Jiang and Liu [23]. Briefly, 5 mL of trichloroacetic acid solution (5% w/v) was combined with 5 mL of a fresh sludge sample and homogenized for 3 min using an ultrasonic homogenizer in an ice-water bath. Following homogenization, 0.5 mL of each homogenized sample was combined with 4.5 mL of 1 × T AE buffer (Tris–acetic acid-EDTA, comprising 40 mmol Tris, 20 mM acetic acid, and 1 mmol EDTA) to produce 5 mL of solution at a final pH of 7.75. Suspended solids were then removed from the sample by filtering through a 0.2-μm membrane. An ATP luminometer (SystemSURE Plus, Hygiene, US) was then used to measure the treated sample, and the luminescence intensity was expressed with relative luminescence units (RLUs). The specific cellular ATP (SATP) was calculated from the cellular ATP (RLU) content/MLSS (mg/L).

### 2.3. EPS Content and Measurement of DNA

A cation exchange resin (CER) strategy modified from Frolund et al. [24] was used to extract extracellular polymeric substance (EPS). The sludge specimens were obtained in reactors with various concentrations of TCS, following which they were centrifuged at 5000 rpm for 15 min. The harvested sludge pellets were rinsed with 100 mmol/L NaCl solution twice. The sludge pellets were then resuspended in phosphate buffer to a pre-established volume. The sludge suspension was then combined with CER in sodium form (Dowex Marathon C, 20–50 mesh, Sigma–Aldrich) at a dosage of 60 g/SS, following which it was stirred at 200 rpm for 10 h at 4 °C. Following this, the specimens were centrifuged at 10,000 rpm and 4 °C for 15 min and filtered through a 0.45-µm cellulose acetate membrane.

### 2.4. Chemicals and Analytical Methods

To calculate the observed biomass yield (Y_obs_), the excess biomass increase was divided by the corresponding decrease in COD concentration after the addition of TCS. Y_obs_ refers to the new amount of sludge generated for each removal of 1 g COD, that is, Y_obs_ = ΔMLSS/ΔCOD. Sludge reduction rate (SR) were determined utilizing Equation (2):

(2) $$SR=\frac{Y_{obs(control)}-Y_{obs(uncoupler)}}{Y_{obs(control)}}\times 100\%$$

Protein measurements followed the modified Lowry method [25,26] using egg albumin as the standard reference. The concentration of polysaccharides was evaluated by the phenol-sulfate acid method. We determined the NH_4_^+^-N, COD, and MLSS using typical methods [27].

The inhibitory actions of TCS on SATP and TTC-ETS activity were assessed based on the corresponding inhibitory rates, which were determined utilizing Equation (3):

(3) $$IR=\frac{R_{0}-R}{R_{0}}\times 100\%$$

where IR is the inhibitory rate (%), R_0_ is the TTC-ETS activity (or SATP) in the control reactor without TCS, and R is the TTC-ETS activity (or SATP) in the TCS reactor.

## 3. Results and Discussion

### 3.1. Sludge Reduction in Activated Sludge Culture with the Addition of TCS

**Figure 1:**
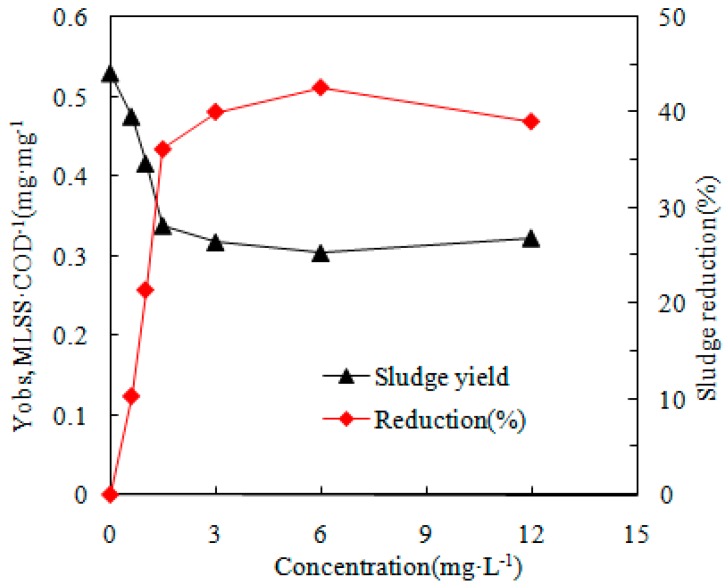
Changes in the sludge yield (Y_obs_) and sludge reduction rate (SR).

The reduction percentages of Y_obs_ at various TCS concentrations are indicated in Figure 1. The TCS content in the present analysis was comparatively higher than that of other studies due to the relatively high MLSS of 2500 mg/L used in comparison with 2000 mg/L used by Li et al. [18]. As indicated in Figure 1, the strength of the chemical uncoupler contributed greatly to the unit biomass and also constituted a more credible indicator due to the ratio of the first uncoupler concentration to the first biomass concentration rather than as a result of the uncoupler content alone, as indicated by Liu [14]. These findings indicate an increase in the reduction percentage of Y_obs_ with TCS concentrations within 0–3 mg/L. The depletion effect on the sludge was noteworthy and reached 40.13% at 3 mg/L, whereas an additional elevation in TCS content did not result in a more significant depletion in Y_obs_. Nearly identical findings have been documented previously [9,14,28].

### 3.2. Pollutant Removal Efficiency in the Presence of TCS

**Figure 2:**
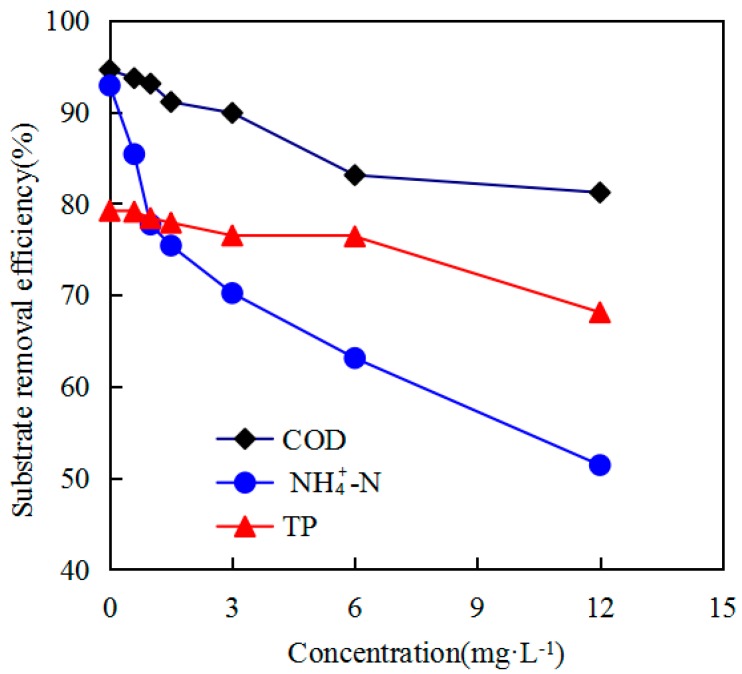
Impacts of metabolic uncouplers on COD elimination, NH_4_^+^-N, and TP elimination at different addition concentrations of TCS.

The COD, TP, and NH_4_^+^-N removal efficiencies under various concentrations of TCS are presented in Figure 2. The results of the impact of TCS on the NH_4_^+^-N removal efficiency show a significant deterioration in the mean removal efficiency for NH_4_^+^-N with the addition of TCS, indicating the significant inhibition of TCS on the nitrification performance. When TCS was 1 mg/L, the average NH_4_^+^-N removal efficiency decreased to 9.1%. However, a high dose of TCS in activated sludge had a remarkable influence on the NH_4_^+^-N removal efficiency, decreasing from 63.1% to 51.4% with an increase in TCS concentration from 6 to 12 mg/L. The findings corroborate those of Zhang et al. [29], who also discovered that NH_4_^+^-N oxidation was greatly obstructed by the addition of uncouplers. This inhibitory impact was the result of the suppression of ammonia-oxidizing bacteria (AOB) activity by metabolic uncouplers, as proven by the decrease in ammonia uptake rate (AUR) with metabolic uncoupler addition [10]. The findings suggest that TCS addition could have a slight lowering impact on wastewater treatment efficiencies. Similar findings have been noted in earlier evaluations [30,31].

At TCS concentrations 0–3 mg/L, slightly lower COD removal efficiencies were detected than in the absence of TCS. A gradual decrease in COD removal efficiencies was observed with increased TCS concentration. TCS concentration 0-6 mg/L had little effect on TP removal, which may have a small effect on polyphosphate accumulating bacteria. TCS concentrations of 6–12 mg/L were associated with COD removal efficiencies of 81%, and they sharply decreased by approximately 8% with the addition of 3 mg/L TCS.

### 3.3. The Mechanisms of Sludge Reduction by TCS

**Figure 3:**
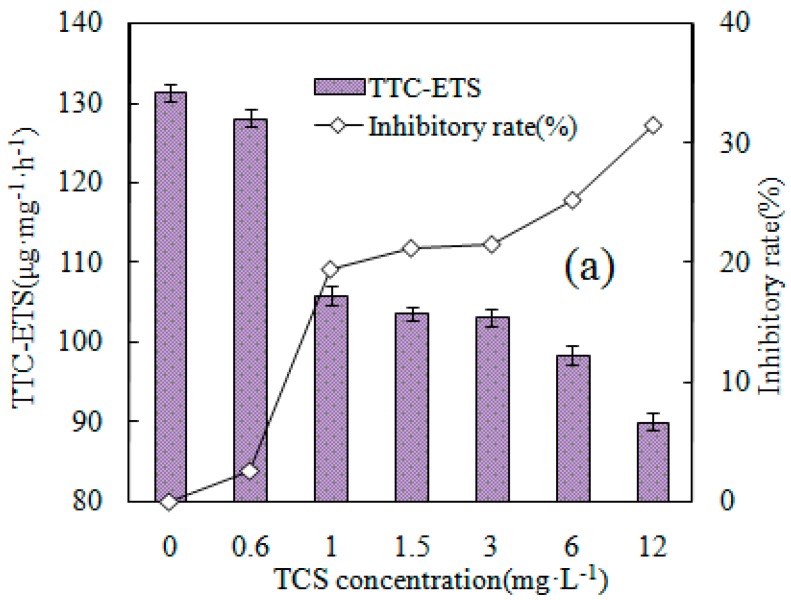

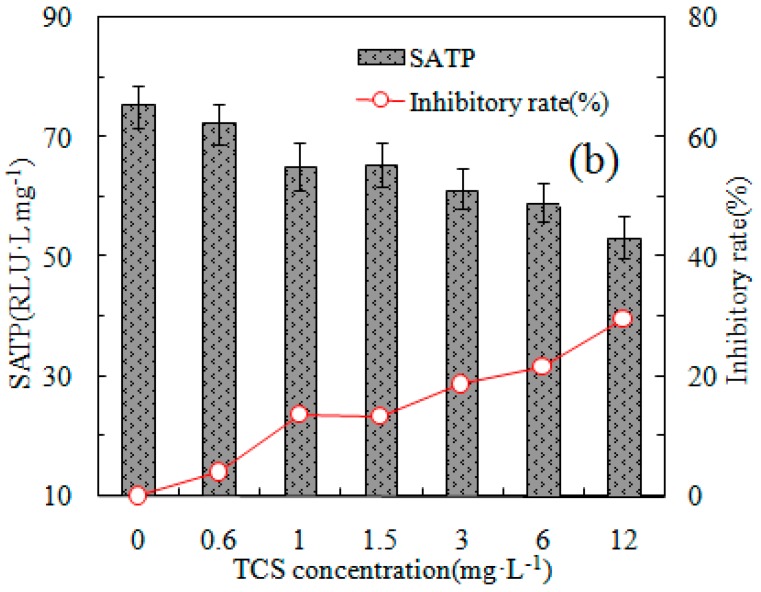
(**a**) The changes in and the inhibitory rates of TTC-ETS at different TCS concentrations; and (**b**) the changes in SATP caused by different concentrations of TCS addition.

**Figure 4:**
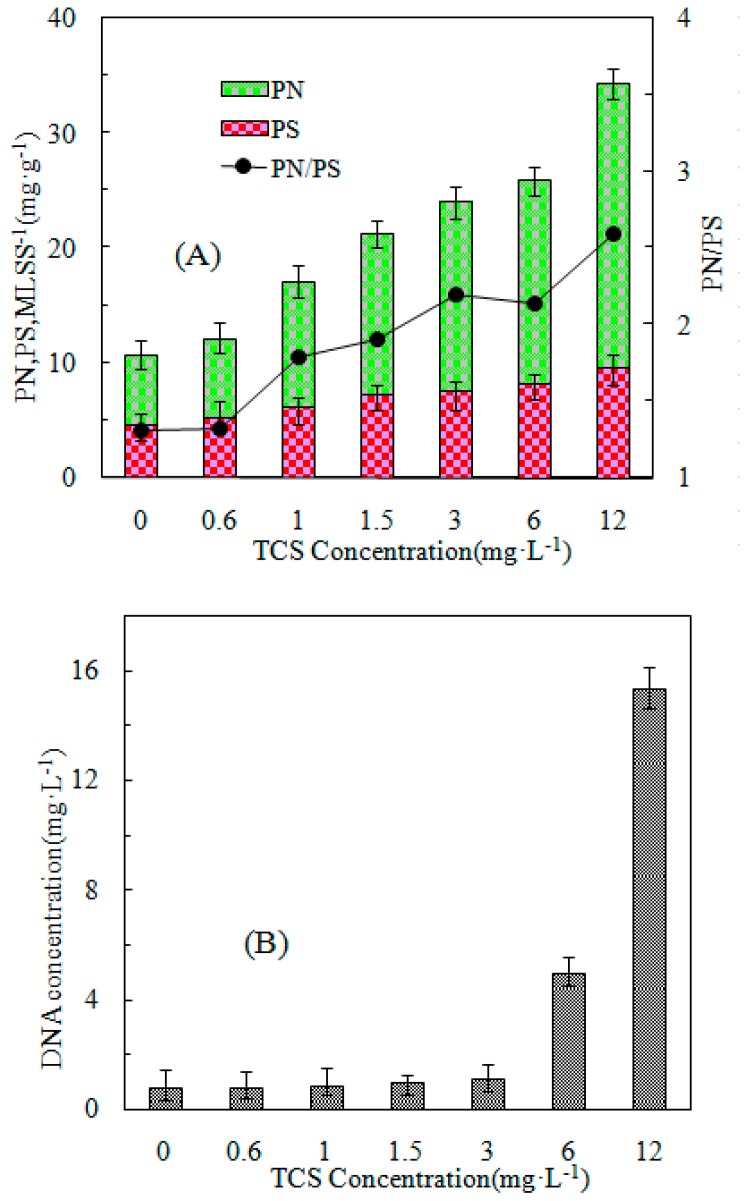
(**A**) Protein/polysaccharide and EPS concentration variations in terms of PN and PS extracted at different TCS concentrations; and (**B**) DNA concentration in the bulk solution at various TCS concentrations.

In the literature, three mechanisms of sludge reduction, namely maintenance metabolism, uncoupled metabolism and lysis–cryptic growth, are discussed. Alterations in ETS activity, SATP, and EPS content in the activated sludge culture as well as the protein and DNA contents in activated sludge supernatants have been widely used in the evaluation of metabolic uncoupling, metabolism, and lysis–cryptic growth [32,33]. We measured these parameters of the activated sludge in this study at different concentrations (0, 0.6, 1, 1.5, 3, 6, and 12 mg/L) of TCS, and the summary of the results is demonstrated in Figure 3 and Figure 4.

ETS activity has been suggested as a particular indicator of aerobic microorganism respiration and as a suitable means of estimating the respiratory possibility of these populations [34]. Typically, this function is quantified by the addition of tetrazolium salt to a biological system. Little information is thus far available on the impacts of TCS on the ETS activity in aerobic sludge microorganisms. TTC was thus used as an activity indicator to comprehensively evaluate the impacts of TCS on ETS function in sludge microorganisms and to determine the alterations in microbial metabolism. Figure 3a indicates the differences in TTC-ETS and the TCS-related inhibitory rates on aerobic sludge microorganisms at various concentrations. With the addition of higher concentrations addition of TCS, lower TTC-ETS activity was found. When the dose of TCS was 0.6 mg/L, little effect on TTC-ETS activity (127.89 µg mg^−1^ h^−1^) was found, only decreasing by 2.54% compare to the control (131.23 µg mg^−1^ h^−1^). With a dose of 1 mg^−1^, the activity sharply decreased to 105.79 µg mg^−1^ h^−1^ (19.38%). At 12 mg/L TCS, the TTC-ETS activity of the microorganisms was 89.98 µg mg^−1^ h^−1^, which indicated a reduction in the sludge of around 31.43% compared to the control.

**Figure 5:**
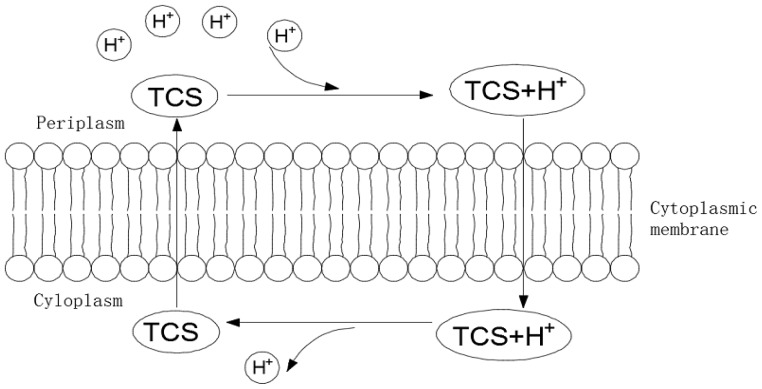
Mechanism of excess sludge reduction induced by TCS.

Uncoupler TCS might promote the cell membrane permeability increase of H+, leading to the decomposition of phosphate coupling oxides, and, subsequently, the synthesis of adenosine triphosphate (ATP) is reduced, thus achieving sludge reduction [12,13]. Figure 5 elaborates the mechanism of excess sludge reduction induced by TCS. The metabolic activity of microbes is dependent on both catabolism and anabolism, which are tightly joined by energy conversion. Following the various biochemical reactions and electron transfers during microbial metabolic processes, additional energy is reserved as ATP. ATP is used in the provision of energy for normal metabolism, reproduction, motility, and nutrient transport. As a result, reductions in the synthesis of ATP would result in a reduction in the energy available in the sludge microorganisms for microbial growth and the successful control of the production of extra activated sludge. The variation in SATP in activated sludge changed very little when the TCS dose was 0.6 mg/L. With the increased addition of TCS, a remarkable reduction in SATP was found. At a TCS dose of 1 mg/L, little difference was detected; however, the reduction was approximately 40% when the TCS concentrations were 6 and 12 mg/L. These findings demonstrate that TCS addition lowered the SATP of the activated sludge and also indicated that the addition of TCS resulted in declines in the TTC–ETS and SATP activities of the activated sludge. The variation in ETS activity demonstrates the impacts of metabolic uncoupling and implies that metabolic uncoupling occurred in all of the TCS-supplemented systems. Furthermore, the variation in ATP directly indicates that metabolic uncoupling had occurred in the microorganisms [32].

The EPS content and composition in the existence of TCS at various concentrations are indicated in Figure 4A. Microorganisms use matrix electrons not only to build active biomass in anabolism but also to produce EPS, which indicates that the addition of TCS could stimulate EPS production. In the present study, dosage with TCS also resulted in increased EPS production with the elevation in TCS concentration from 0 to 12 mg/L. The outcomes corroborate those of Li et al. [18]. With the difference in TCS dosage, the EPS protein content increased sharply to 18.71 mg/g SS, while the EPS polysaccharide level changed only slightly to 4.91 mg/g SS. The results suggest that TCS addition stimulated more protein production than polysaccharide production. This agrees with the findings of Li et al. [18]. Alterations in the EPS content and composition in the presence of TCS imply that cells are sensitive to the existence of metabolic uncouplers and thus increase their EPS production to safeguard active biomass for improved survival under suboptimal environments [10,29].

The DNA levels in bulk solution are indicative of the degree of cell lysis (Aquino and Stuckey 2004). As indicated in Figure 4B, the DNA concentration in the supernatant in the absence of TCS only measured 1 mg/L, which suggests a slight degree of cell lysis. The TCS concentrations of 0.6–3 mg/L were associated with a decreased level of DNA (< 2.0 mg/L). However, an elevation in the TCS concentration to 6 and 12 mg/L resulted in a sharp increase in the DNA level to 4.97 and 15.34 mg/L, respectively, suggesting increased cell lysis at these high TCS concentrations.

## 4. Conclusion

(1) The degree of sludge reduction was dependent on the ratio of the first uncoupler concentration to the first biomass concentration, which was MLSS:TCS of 2500:3 mg/L.

(2) TCS at low concentrations had little effect on TP removal, followed by COD, but it did have a larger effect on the elimination of NH_4_^+^-N by TCS.

(3) The presence of TCS can weaken TTC-ETS activity and reduce SATP synthesis, leading to inhibition of microbial activity.

(4) A difference in concentration was the demonstrated mechanism of sludge reduction. High concentration (6 and 12 mg/L) involved lysis–cryptic growth in sludge reduction by TCS. At a slight concentration (≤3 mg/L), metabolic uncoupling and alterations in energy distribution were pivotal for achieving effective sludge reduction by TCS.

## Figures and Tables

**Table 1:** Constituents of the synthetic wastewater solution added to the sequencing batch reactors.

Constituents	Concentration (mg L^−1^)	Constituents	Concentration (mg L^−1^)
Sucrose	331.6	FeCl_2_2H_2_O	3.3
CH_2_COONa	215.3	MgCl_2_6H_2_O	3.4
NH_4_Cl	175	MnSO_4_	0.051
KH_2_PO_4_	3.69	CoSO_4_	0.038
CaCl_2_2H_2_O	3.4	CuSO_4_	0.071
